# The Book World of Medicine and Science

**Published:** 1899-11-04

**Authors:** 


### THE BOOK WORLD OF MEDICINE AND SCIENCE.

The Universal Illusion of Free Will and Criminal Responsibility. By A. Hamon, of the New University, Brussels. (London: University Press. 1899. Large octavo. Pp. 138. Price 3s. 6d.)

The battle of metaphysicians is a perennial one. The combatants enter the lists, give and receive ringing blows on their steel-clad heads, retire gracefully, and come up again smiling when time is called. They have continued this game for two thousand years, quite forgetting that a long dispute signifies that both parties are wrong. It is time to give it up, at least as a serious occupation, although there would be no harm in every well-conditioned stripling passing through an attack of metaphysics, just as he does through other infantile diseases, and so preserve an immunity for the rest of his life.

This treatise of Hamon's is intended to lead up to criminal responsibility, or rather irresponsibility, and after showing to his own satisfaction that the will is not free, he defines crime in the following words: "Crime is every conscious act which injures the liberty of action of a similar individual of the same species." The incompleteness of this definition is manifest; but it may be passed over, for if the will be not free it is difficult to see how there can be any crime at all in the ordinary acceptation of the term, and the word "responsibility" may be left out of our vocabularies, as it and free-will must sink or swim together. The Stoics believed in free-will, and they did not often deceive themselves. The early Christian Fathers like Justin Martyr and Irenæus also believed it, and it must have been no easy matter for them to reconcile free-will with Divine foreknowledge. It may be that the will is merely a psychological ghost generated at the moment consciousness passes into action; and whether the mind be free or fettered or the will merely a product of consciousness matters not to the man in the street. If he detect a thief in the act of stealing his watch he hands him over to the police, and neither the thief nor his advocate can make any impression on the judge by saying, or even proving, that the prisoner was impelled by an inexorable fate to commit the theft. Punishment would follow, unless the plea of insanity could be sustained.

In our opinion the best part of the book, and certainly the most interesting to the physician, is that section which deals with the responsibility of the insane. The views held by the jurists of France, Germany, Spain, and England are very clearly stated and commented on. Article 64 of the French Code says: There is no crime or fault when the accused was in a state of insanity at the time of the act, or when he has been constrained by a force which he was not able to resist. This, which we believe to be part of the Code Napoleon, is very well expressed; and the author points out that the German code is even more explicit, for it is necessary that the accused should have had liberty of will, otherwise there is no crime. Again the Spanish code declares that the imbecile, the insane, and the unbalanced in mind are not responsible. Here would step in the difficulty of defining the words "imbecile" and "insane." The English judicial idea of responsibility or of irresponsibility consists in the presence or absence of a knowledge of right and wrong. This definition is diametrically opposed to the views of every alienist physician. If it did not lead to such sad results it would be amusing, and we hope the day is not far off when, in Hamon's words, "public opinion aroused by the scientists will oblige the British judges to take account of scientific discoveries."

When the investigations pass beyond the clear practical bearings which they possess when considering the responsibility of individuals alleged to be insane, we cannot say we are able to see that much good is likely to be derived from these speculations. Freewill will have its Aristotles, its Kants, and its Hamiltons; and Necessity will have its Spinozas, its Hobbes, its Mills, and its Shelleys as long as the world lasts. But the plain men of the earth will heed none of these, and they will continue to say that a man, that is, a sane man, acts with free will when he "voluntarily and intentionally performs an action," and if it be a criminal action he will be held responsible for it.

Orthopædic Surgery. A Text-book of the Pathology and Treatment of Deformities. By J. Jackson Clarke, M.B.Lond., F.R.C.S. (London: Cassell and Co. 1899. With 309 illustrations. Price 21s.)

This admirable book is a very complete and instructive treatise on a subject upon which much ignorance prevails. No one can take up the study of orthopædics without seeing how largely some of the serious deformities we meet with in practice are due to oversight or neglect in their earlier stages, and the comparatively slight attention which the subject often receives in the ordinary surgical course renders it all the more necessary that the student and the practitioner (who in such a subject must often also be a student) should have at hand a treatise dealing with orthopædics as a whole. Mr. Jackson Clarke devotes a considerable amount of space to the consideration of splints, bandages, and apparatus of various kinds, not only laying down the principles to be borne in mind in their application, but describing very fully how they should be applied to the treatment of individual cases. In fact, the keynote to the book is the individuality of the case and the necessity of the surgeon taking it in hand and dealing with its every phase himself, employing the instrument-maker only for his proper work as a maker of instruments—not as is so often done as an expert in the treatment of deformities. It is unfortunately the fact that either from defective early training, from dislike to all such details, or maybe because their brains are not built that way, some surgeons seem quite "out of it" in regard to all "merely" mechanical questions, and such had better hand over the treatment of deformities to others; but to a man who can appreciate mechanical problems, few things can be more interesting than orthopædic work, and to such work this book will serve as a most useful guide. The chapters on tuberculous, or, as the author calls it, "tubercular" disease of the hip and of the spine, are full and safe guides to the treatment of these very difficult cases. On the question of forcible straightening of the deformity in tubercular spondylitis Mr. Jackson Clarke holds to the view that cases treated from the beginning by efficient mechanical means will give as good results as those treated by forcible extension, and that only early cases free from complications, such as abscess, &c., would be suitable for the operation, even should it prove to be successful in diminishing or removing the deformity. A good account is given of congenital dislocation of the hip. On the other hand, the description of scoliosis and its treatment, although fairly full, is somewhat disappointing. On the whole, however, the book will be found very useful as giving a good account of the present position of orthopædic surgery.

